# Assessment of self-medication practices and safety profile of medicines utilisation among pregnant women attending antenatal clinics in Freetown, Sierra Leone: a multicentre cross-sectional study

**DOI:** 10.1080/20523211.2024.2380874

**Published:** 2024-07-24

**Authors:** Onome Thomas Abiri, Shakiratu Lawal, Joshua Coker, James Baligeh Walter Russell, Ibrahim Franklyn Kamara, N’falie Ibrahim Sesay, Joseph Sam Kanu, Foday Umaro Turay, Michael Lahai, Henry Edward Clarence Carter, Mohamed Bawoh, Mohamed Samai

**Affiliations:** a Department of Pharmacology and Therapeutics, College of Medicine and Allied Health Sciences, University of Sierra Leone, Freetown, Sierra Leone; b Department of Clinical Pharmacy, College of Medicine and Allied Health Sciences, University of Sierra Leone, Freetown, Sierra Leone; c Department of Internal Medicine, College of Medicine and Allied Health Sciences, University of Sierra Leone, Freetown, Sierra Leone; d Reproductive Maternal Newborn, Child and Adolescent Health, Universal Health Coverage/Life Course Cluster, World Health Organisation Country Office, Freetown, Sierra Leone; e Department of Obstetrics and Gynaecology, University of Sierra Leone Teaching Hospital Complex, Freetown, Sierra Leone; f Department of Community Medicine, College of Medicine and Allied Health Sciences, University of Sierra Leone, Freetown, Sierra Leone; g Department of Pharmaceutical Chemistry, College of Medicine and Allied Health Sciences, University of Sierra Leone, Freetown, Sierra Leone; h Department of Pharmaceutics, College of Medicine and Allied Health Sciences, University of Sierra Leone, Freetown, Sierra Leone

**Keywords:** Self-medication, pregnancy, predictors, antenatal clinic, teratogenic effects, Sierra Leone

## Abstract

**Background:**

Despite the potential foetal and maternal risks of self-medication, studies on self-medication practice and the safety profile of medicines used during pregnancy are scarce in our setting. This study determined the self-medication practice and safety profile of medicines used among pregnant women.

**Methods:**

This cross-sectional study was conducted in face-to-face interviews among 345 pregnant women at three hospitals in Sierra Leone. Data were analysed using descriptive statistics and binary logistic regression to determine the prevalence and associated factors of self-medication.

**Results:**

A total of 345 pregnant women participated in the study. The prevalence of self-medication prevalence among pregnant women with conventional and/or herbal medicine was 132 (38.3%). Also, 93 (75%) of the conventional medicines (CMs) were categorised as probably safe, of which paracetamol 36 (29.0%) was commonly used, followed by amoxicillin 23 (18.5%) and antimalarials 22 (17.7%) for common illnesses such as headache 30 (25.4%), urinary tract infection 23 (19.4%) and malaria 22 (18.6%). The most common reason for self-medication was previous experience with the disease 24 (27.3%). *Luffa acutangula* 19 (30.2%) was the most used herbal medicine (HM), and Oedema 30 (47.6%) was the most reported ailment. Among the HM users, 34 (54.0%) believe they are more effective than CMs. Secondary school education (AOR = 2.128, 95%CI = 1.191–3.804, *p* = 0.011), tertiary education (AOR = 2.915, 95%CI = 1.104–7.693, *p* = 0.031), monthly income of greater than NLe 1,000 (AOR = 4.084, 95% CI = 1.269–13.144, *p* = 0.018), and perceived maternal illness (AOR = 0.367, CI = 0.213–0.632, *p* = <0.001) were predictors of self-medication.

**Conclusion:**

Self-medication practice was highly prevalent and was associated with educational status, monthly income, and perceived maternal illness during pregnancy. Therefore, intervention programmes should be designed and implemented to minimise the practice and risk associated with self-medication among pregnant women.

## Introduction

Self-medication is described as the use of medicines without consulting a clinician to treat self-diagnosed symptoms or illnesses (WHO, 2000). Women may encounter ailments such as headaches, pain, and urinary tract infections during pregnancy and may self-medicate to relieve these symptoms despite concerns for both the foetus and the mother (Marwa et al., 2018). This practice has been reported in both developed and developing countries, with potentially serious adverse effects on the foetus and mother, including low birth weight, premature birth, respiratory problems, and mortality (Mohseni et al., 2018; Ross et al., 2015).

Concurrent use of conventional medicines (CMs) and herbal medications (HMs) by pregnant women can pose potential risks to both the mother and the foetus, as the ingredients and safety of HMs have not been well characterised (Adane et al., 2020). This is particularly concerning in low-income countries, such as Sierra Leone, where access to quality antenatal services, medical supplies, poverty, and illiteracy are significant issues (Limaye et al., 2017; Parulekar et al., 2016). As medicines are poorly regulated and readily available from most pharmaceutical outlets, pregnant women can access them easily and may only seek proper medical attention when complications arise (Adanikin & Awoleke, 2017).

Previous studies have identified common reasons for self-medication, including the urge for self-care for minor illnesses, previous experience with the disease, time constraints, inaccessibility to healthcare professionals, high consultation fees, distance from health facilities, and exposure to drug advertisements (Mohseni et al., 2018; Rahmani et al., 2019). Other studies have identified factors such as age, occupation, education, income level, previous self-medication, and gestational age as predictors of self-medication (Shokrzadeh et al., 2020; Zewdie et al., 2018). However, safety data for medications prescribed during pregnancy are limited owing to women's exemption from clinical trials and the lack of data available from regulators, manufacturers, and pre-clinical studies (Krubiner et al., 2021; Stock & Norman, 2019). Although different medicine classification systems, such as the Australian and United States methods, can categorise medications as safe or contraindicated based on foetal risks during pregnancy, these schemes do not cover all medications, creating a quagmire for clinicians and consumers during prescribing, dispensing, and administration (Nordeng, 2016).

Previous studies in Sierra Leone have examined self-medication with CMs among non-pregnant adults, undergraduate students, and inpatient CMs prescription patterns during pregnancy (Abiri et al., 2021; Atolagbe et al., 2020; Johnson et al., 2022; Moseray et al., 2024). Furthermore, although data on HM self-medication among pregnant women have been reported by James et al. (2018a), their study was not multicentre and did not explore CM self-medication. Therefore, this study aimed to assess self-medication practices and predictors among pregnant women attending antenatal clinics in three government hospitals in Freetown, Sierra Leone and to determine the safety profiles of these medicines concerning their potential risks to the foetus.

## Methods and materials

### Study design and setting

This was a multicentre cross-sectional descriptive study conducted from April 1 to July 31, 2022, at the antenatal clinics of the Lumley Government Hospital (LGH), King Harman Maternity and Children Hospital (KHMCH) and Rokupa Government Hospital (RGH). The hospitals, located in the Western, Central, and Eastern parts of the city, provides comprehensive emergency obstetric and newborn care, as well as inpatient and outpatient paediatric and maternity services.

### Study population

Consenting pregnant women visiting antenatal clinics at LGH, RGH, and KHMCH, regardless of age were included in the study.

### Sample size determination and sampling

The sample size was determined using Fisher's formula for single-population studies, where *p* = proportion of pregnant women who self-medicated (69%) according to a previous study (Gbagbo & Nkrumah, 2020), z = confidence level at 95%, and d = degree of precision of 5%. The sample size was 329. The sample size used for this study was 345, after considering potential issues such as non-response, which could affect the validity and reliability of the results. Based on the probability proportional to size sampling, the sample sizes for the individual hospitals were 148, 136, and 61 for KHMCH, LGH, and RGH, respectively. Consecutive sampling was used to obtain sampling units at the study sites.

### Data collection procedure and tools

Data were collected using a validated structured questionnaire from previous studies (Mohseni et al., 2018; Sema et al., 2020). The questionnaire was divided into four sections, with the first containing sociodemographic information about the participants. The second section focused on obstetric information such as gravidity and gestational age. Sections three and four asked about self-medication practices with CM and HM, including the reasons and conditions for self-medication and the types of medication used. The questionnaire was pretested in a none participating hospital for content and validity, and the final version was developed based on feedback. The data was collected through face-to-face interviews with consented participants between 1 May and 31 May, 2022.

### Data analysis

After data collection, the data were cleaned, coded, and analysed using Statistical Package for Social Sciences version 20 (IBM Statistics, Armonk, NY, USA). The results were presented as mean and standard deviations for continuous variables, and counts and percentages for other variables. The dependent variable was self-medication with conventional and/or herbal medicines, while the independent variables included age, marital status, educational status, occupation, monthly income, gestational age, gravidity, perceived maternal illness at the time of the interview, and time on foot from home to the nearest hospital. Far was defined as two hours and more on foot, not too far as one to two hours on foot, and near as less than one hour on foot.

Pearson Chi-square was used to analyse the association between the dependent and independent variables, and univariate and multivariate logistic regression analyses were conducted to identify predictors of self-medication. Independent variables with a *p*-value ≤0.2 in the univariate model were included in the final multivariate model. The final model was assessed using Hosmer-Lemeshow’s goodness-of-fit test, which indicated a good fit with the data (*p* = 0.394). Statistical significance was set at *P* < 0.05.

The United States Food and Drug Administration (USFDA) and Australian Therapeutic Goods Administration (AU-TGA) were used to classify CMs based on foetal risk (Nordeng, 2016). The AU-TGA categorisation system, which uses letters (A, B1, B2, B3, C, D, and X) to define medicine safety, was employed as the primary approach if the USFDA categorisation did not cover the medicine. The USFDA categorisation, consisting of five classes (A, B, C, D, and X), where Category A indicates the safest medicine and Category X indicates teratogenic medications, was used as the secondary method. Previous studies categorised medicine exposures into ‘probably safe,’ ‘potentially risky,’ or ‘unclassified’ classes of more clinical interest (Rouamba et al., 2018; Trønnes et al., 2017). For combination medicines and those with several active ingredients, the risk was categorised based on the dominant active ingredient and the ingredient with the highest risk. The ‘probably safe’ medicines category comprised AU-TGA categories A, B1, and B2, and USFDA categories A and B. The ‘potentially risky’ group consisted of AU-TGA categories B3, C, D, and X, and USFDA Categories C, D, and X. Medicines were classified into therapeutic classes according to the Anatomical Therapeutic Chemical Classification System (WHO, 2021).

## Results

### Socio-demographic and obstetric characteristics of participants

Among the 345 pregnant women who were invited to participate in the study, all agreed to take part with a 100% response rate. The mean age (standard deviation) of the participants was 27.5 (4.6) years. As shown in Table 1, most participants were aged between 26 and 36 years, married, had a secondary school education, were privately employed, and earned a monthly income of more than NLe 1,000. Additionally, most of the women lived within 1–2 hours of the nearest health facility, were multigravida, in their third trimester, and reported feeling ill during the interview. Education, monthly income, and perceived illness were significantly associated with self-medication, with *p*-values of 0.005, 0.039, and <0.001, respectively.

**Table 1.** Characteristics of study participants attending antenatal clinics at LGH, RGH, and KHMCH in Freetown, 2022 (n = 345).

	Self-medication				
Characteristics	Yes n (%)	No n (%)	Total n (%)	*Chi-square*	*p-value*
**Age**					
15–25	46 (34.8)	70 (32.9)	116 (33.6)	0.811	0.667
26–36	85 (64.4)	139 (65.3)	224 (64.9)		
37–47	1 (0.8)	4 (1.9)	5 (1.4)		
**Marital Status**					
Single	55 (41.7)	77 (36.2)	132 (38.3)	4.481	0.306
Married	77 (58.3)	136 (63.8)	213 (61.7)		
**Educational Status**					
No education	40 (30.3)	34 (16.0)	74 (21.4)	12.806	**0**.005*
Primary School	8 (6.1)	13 (6.1)	21 (6.1)		
Secondary School	68 (51.5)	118 (55.4)	186 (53.9)		
Tertiary	16 (12.1)	48 (22.5)	64 (18.6)		
**Occupation**					
Student	5 (3.8)	10 (4.7)	15 (4.3)	6.814	0.146
Civil Servant	6 (4.5)	20 (9.4)	26 (7.5)		
Private Employment	62 (47.0)	102 (47.9)	164 (47.5)		
House Wife	14 (10.6)	10 (4.7)	24 (7.0)		
**Monthly Income**					
Less than NLe 1,000	74 (56.1)	95 (44.6)	169 (49.0)	4.283	**0**.039*
Greater than NLe 1,000	58 (43.9)	118 (55.4)	176 (51.0)		
**Time to the Nearest Hospital**					
Near (< 1 hour on foot)	34 (25.8)	38 (17.8)	72 (20.9)	3.094	0.213
Not too far (1-2 hours on foot)	64 (48.5)	114 (53.5)	178 (51.6)		
Far (> 2 hours on foot)	34 (25.8)	61 (28.6)	95 (27.5)		
**Gravity**					
Primigravida	46 (34.8)	80 (37.6)	126 (36.5)	0.258	0.611
Multigravida	86 (65.2)	133 (62.4)	219 (63.5)		
**Gestational Age**					
First trimester	4 (3.0)	8 (3.8)	12 (3.5)	2.095	0.351
Second trimester	58 (43.9)	77 (36.2)	135 (39.1)		
Third trimester	70 (53.0)	128 (60.1)	198 (57.4)		
**Perceived Maternal Illness**					
No	25 (18.9)	80 (37.6)	105 (30.4)	13.344	**<0**.001*
Yes	107 (81.1)	133 (62.4)	204 (69.6)		

NLe (New Leones), Lumley Government Hospital (LGH), Rokupa Government Hospital (RGH), King Harman Road Maternity and Children Hospital (KHMCH), Bold figures represent statistically significant findings (*p* < 0.05)*.

### Prevalence of self-medication among pregnant women

Of the 345 pregnant women interviewed, 132 (38.3%) practiced self-medication using conventional or herbal medicines. Of the 132 (38.3%) women who practiced self-medication, 88 (25.5%) used CM, and 63 (18.2%) used HM.

### Reasons for use and characteristics of self-medication with conventional medicines among pregnant women

The commonest medical condition that caused self-medication with conventional medicine was headache 30 (25.4%). Previous experience with the disease 24 (27%) was the most common reason for using CMs. Regarding how they got the medicines, 67 (76%) self-ordered from a pharmacy, and the self-experience of participants 46 (13.3%) was the most used source of.

information. Only 12 (14%) of the CM users usually read the patient information leaflet (PIL), and 9 (75%) generally read about the dosage. Among those who did not usually read the PIL, 28 (37%) said they did not know how to read it (Table 2).

**Table 2.** Prevalence and pattern of self-medication among pregnant women using conventional medicines at the LGH, RGH, and KHMCH in Freetown, 2022.

Variable		n (%)
Reasons for self-medication	Save time	2 (2.3)
	Previous experience with the disease	24 (27.3)
	Lack of access to physician	7 (8.0)
	High cost of a visit to the physician	12 (13.6)
	Costly medical expense	9 (10.2)
	The disease is simple; no need to see a doctor	20 (22.7)
	Long distance to the health facility	5 (5.7)
	Not yet joined a clinic	9 (10.2)
Conditions that caused self-medication	Headache	30 (25.4)
	Urinary tract infection	23 (19.4)
	Malaria	22 (18.6)
	Pain	19 (16.9)
	Heartburn	13 (11.0)
	Morning sickness	10 (8.5)
How did you get medicines?	Taken from friends	10 (11.0)
	Self-ordered from the pharmacy	67 (76.0)
	Taken from a neighbour	2 (2.0)
	I take medicines available at home	9 (11.0)
Source of information for self-medication	Your experience	46 (13.3)
	Advice from a friend	15 (4.3)
	Advice from a relative	13 (3.8)
	Advice from a nurse	11 (3.2)
	Advice from a pharmacist	3 (0.9)
Usually, read the patient leaflet	Yes	12 (13.6)
	No	76 (86.4)
Information got from the leaflet	Dosage	9 (75.0)
	Indication	3 (25.0)
Why don’t you read the leaflet?	I don't know how to read	28 (36.8)
	I don't understand the information on the leaflet	22 (29.0)
	Drugs don't have a leaflet	26 (34.2)
Why are you not self-medicating?	Self-medication may harm the foetus	82 (31.9)
	Self-medication may harm the mother and foetus	29 (11.3)
	Informed by the doctor not to practice self-medication	146 (56.8)

Lumley Government Hospital (LGH), Rokupa Government Hospital (RGH), King Harman Road Maternity and Children Hospital (KHMCH).

### Reasons for use and characteristics of self-medication with herbal medicines among pregnant women

Table 3 shows that *Luffa acutangula* (Rabena) was commonly used for ailments (30.2%), followed by *Citrus aurantifolia* (Lime leaf) [25.4%]. Oedema was the most reported ailment (47.6%). Users mainly received information about herbal medicines (HM) from family and friends (73.0%). Pregnant women usually prepare HM themselves (47.6%) and believe that they are more effective than conventional medicines (54.0%). However, 122 (43.3%) of those who did not use HM stated that they did not like HMs.

**Table 3.** Prevalence and pattern of self-medication with herbal medicines among pregnant women attending antenatal clinics at the LGH, RGH, and KHMCH in Freetown, 2022.

Variable		n (%)
Types of herbs used	Rabena (*Luffa acutangula*)	19 (30.2)
	Lime leaf (*Citrus aurantifolia*)	16 (25.4)
	Scratch leaf (Ficus exasperata)	5 (7.9)
	Kola leaf (Garcinia kola)	2 (3.2)
	Unable to specify	21 (33.3)
Reasons for herbal medicine use	Herbal medicines have a lower cost	21 (33.3)
	Herbal medicines are more effective than conventional medicines	34 (54.0)
	Unable to specify	8 (12.7)
Ailments for which HMs were used	Oedema	30 (47.6)
	Vomiting	20 (31.7)
	Infection (unspecified)	7 (11.1)
	Malaria	6 (9.5)
Source of information	Family and friends	46 (73.0)
	Traditional healers	15 (28.3)
	Neighbours	2 (3.2)
Where did you get the HM from?	Self-preparation	30 (47.6)
	Traditional healers/herbalists	12 (19.0)
	Traditional birth attendants	3 (4.8)
	Family	18 (28.6)
Why are you not self-medicating?	Herbal medicine may harm the foetus	103 (36.5)
	Herbal medicine may harm cause abortion	2 (0.7)
	Herbal medicine may harm the mother and foetus	48 (17.0)
	I don't have information about the proper dosage of herbal medicine	7 (2.5)
	I don't like herbal medicines.	122 (43.3)

Lumley Government Hospital (LGH), Rokupa Government Hospital (RGH), King Harman Road Maternity and Children Hospital (KHMCH).

### Safety classification of medicines used in pregnancy

Table 4 shows that 93 (75.0%) of the CMs used by pregnant women were classified as probably safe using the US FDA and AU-TGA classification methods based on foetal risk, with paracetamol 36 (29%), followed by amoxicillin 23 (18.5%) as the commonly self-medicated medicines. In addition, 31(25.0%) of the medications were categorised as potentially risky to use during pregnancy, including artemether-lumefantrine 22 (17.7%), followed by ibuprofen 9 (7.3%). For Rabena and Lime leaf, existing literature reveals that they may have harmful effects on the foetus.

**Table 4.** AUTGA and USFDA modified medicine risk classification.

			Trimester					
Medicines used	n (%)	ATC code	1	2	3	AU-TGA category	US FDA category	Risk category
Paracetamol	36 (29%)	N02BE01	1	19	16	A	NA	Probably safe
Amoxicillin	23 (18.5%)	J01CA04		12	11	A	B	Probably safe
Artemether/Lumefantrine	22 (17.7%)	P01BF01	1	11	10	D	NA	Potentially risky
Aluminum/Magnesium hydroxide	13 (10.5%)	A02AD02	-	8	5	Exempted	NA	Probably safe
Oral Rehydration Salt	10 (8.1%)	A07CA	1	9	-	Exempted	NA	Probably safe
Ibuprofen	9 (7.3%)	G02CC01	-	5	4	C	NA	Potentially risky
Multivitamins	4 (3.2%)	A11AA05	1	3	-	Exempted	NA	Probably safe
Folic acid	7 (5.6%)	B03BB01	-	4	3	A	A	Probably safe

NA – Not assigned, USFDA – United States Food and Drug Administration, AU-TGA – Australian Therapeutic and Goods Administration, ATC – Anatomical Therapeutic Chemical classification.

### Predictors of self-medication among pregnant women

The multivariate analysis showed that secondary school education (AOR = 2.128, 95%CI = 1.191–3.804, *p* = 0.011), tertiary education (AOR = 2.915, 95%CI = 1.104–7.693, *p* = 0.031), monthly income of greater than NLe 1,000 (AOR = 4.084, 95% CI = 1.269–13.144, *p* = 0.018), and perceived maternal illness (AOR = 0.367, CI = 0.213–0.632, *p* = <0.001) were also shown to be statistically significant (Table 5). Furthermore, pregnant women with secondary school education, tertiary education, and monthly income greater than 1,000 were 2.1, 2.9, and 4 times more likely to practice self-medication during the current pregnancy. Additionally, the odds of self-medication practice among pregnant women with no perceived illness was about 0.36 times lesser than those who perceived to be ill.

**Table 5.** Predictors of self-medication among pregnant women with CMs and HMs.

Characteristics	COR (95%Cl)	*p*-value	AOR (95% CI)	*p*-value
**Age**				
15–25	1			
26–36	1.075 (0.679–1.702)	0.759		
37–47	2.629 (0.285–24.267)	0.394		
**Marital Status**				
Single	1			
Married	1.262 (0.809–1.968)	0.306		
**Educational Status**				
No education	1		1	
Primary School	1.912 (.709 - 5.157)	0.201	2.035 (0.718–5.763)	.181
Secondary School	2.042 (1.183–3.524)	**0**.010*	2.128 (1.191–3.804)	.011*
Tertiary	3.529 (1.705–7.305)	**0**.001*	2.915 (1.104–7.693)	.031*
**Monthly Income**				
Less than NLe 1,000	1		1	
Greater than NLe 1,000	1.585 (1.024–2.454)	**0**.039*	4.084 (1.269–13.144)	**0**.018*
**Occupation**				
Unemployment	1		1	
Student	1.667 (0.407–6.818)	0.477	0.399 (0.063–2.535)	0.330
Civil Servant	0.823 (0.269–2.518)	0.732	0.356 (0.062–2.035)	0.245
Private Employment	0.357 (0.093–2.746)	1.372	0.473 (0.98–2.291)	0.353
House Wife	0.789 (0.253–2.458)	0.683	1.115 (0.295–4.215)	0.873
**Gravity**				
Primigravida	1			
Multigravida	0.889 (0.565–1.399)	0.611		
**Gestational Age**				
First trimester	1		1	
Second trimester	0.664 (0.191–2.311)	0.520	0.673 (0.170–2.673)	0.574
Third trimester	0.914 (0.266–3.144)	0.887	0.991 (0.251–3.914)	0.990
**Perceived Maternal Illness**				
Yes	1		1	
No	0.388 (0.232 – .651)	**<0**.001*	0.367 (0.213–0.632)	**<0**.001*
**Time to the Nearest Hospital**				
Near (< 1 hour on foot)	1			
Not too far (1-2 hours on foot)	1.594 (0.915–2.776)	0.100		
Far (> 2 hours on foot)	1.605 (0.860–2.998)	0.137		

Bold figures represent statistically significant findings (*p* < 0.05)*, COR – crude odd ratio, AOR – adjusted odd ratio, CI – confidence interval.

## Discussion

We conducted a study in three government hospitals in Freetown, Sierra Leone, to investigate self-medication practices and safety profiles of conventional medicines among pregnant women attending antenatal clinics. Our findings showed that a significant proportion of pregnant women self-medicated with conventional and/or herbal medicines, with most categorised as probably safe. Factors such as educational status, monthly income, and perceived maternal illness were associated with self-medication practices.

Over one-third of the pregnant women in our study (38.3%) self-medicated during their current pregnancy, which is consistent with studies conducted in Northern Jordan and Ethiopia (Alsous et al., 2021; Sema et al., 2020). This might be due to the poor medicine regulatory system and weak enforcement in resource-limited settings, such as Sierra Leone, making it easier for pregnant women to access medicines without a prescription, despite the potential hazards associated (Sah et al., 2020). Additionally, cultural beliefs may lead pregnant women to prefer traditional or herbal therapies to medical advice from professionals (Akhagba, 2017). Although the study was conducted in Freetown, where access to antenatal care services is free and should not be a problem, healthcare facilities may sometimes experience a shortage of medical supplies, predisposing pregnant women to self-medication (Limaye et al., 2017).

The prevalence of self-medication in this study was higher than that in Mexico but lower than that in Ghana (Alonso-Castro et al., 2018; Gbagbo & Nkrumah, 2020). Factors contributing to the variation included differences in awareness of the risks of self-medication, population demographics, and study design (Adane et al., 2022). It is crucial for pregnant women to understand that self-medication can pose significant risks to their unborn child, and necessary measures, such as raising awareness and enforcing regulations, should be taken to prevent it. In this study, previous experience with the disease and belief that the disease is simple were common reasons for self-medication with conventional medicine. While previous pregnancies may have provided women with knowledge and experience, this does not mean that the medicines were risk-free (Mohseni et al., 2018). Therefore, health care providers should provide personalised counselling to pregnant women regarding the potential adverse effects of self-medication on their unborn children.

Previous studies have shown that herbal medicines are preferred to conventional medicines because of their perceived effectiveness, which may be attributed to personal experiences and recommendations from family and friends, as indicated in our study (James et al., 2018a). Similar to previous studies, herbal medicines were mostly self-prepared, posing a risk of adverse pregnancy outcomes owing to the lack of evidence-based standardisation for administering herbal medicines (Jambo et al., 2018). Furthermore, herbal medicines may interact with drugs owing to their inherent toxicity and diverse ingredients, resulting in altered pharmacokinetics, pharmacodynamics, and potentially harmful outcomes (Asokkumar & Ramachandran, 2020).

Consistent with previous studies, most CMs used by pregnant women, including paracetamol and amoxicillin, were classified as probably safe and obtained from pharmacies; headache, urinary tract infection, and malaria were identified as the most common indications for use (Girmaw et al., 2023; Marwa et al., 2018; Rouamba et al., 2018). Paracetamol, the most frequently used medication, poses no known teratogenic or toxic risk to the foetus; however, maternal or foetal liver damage may occur at toxic doses (Kamath et al., 2021). However, prenatal exposure to paracetamol is associated with attention-deficit hyperkinetic disorder, asthmatic symptoms, and impaired psychomotor development during early childhood (Liew et al., 2021). Therefore, caution should be exercised when administering paracetamol during pregnancy. Furthermore, the use of amoxicillin in this context is considered irrational because it can lead to the development of antibiotic resistance, resulting in lengthy hospital stays, financial losses, and mortality (Rather et al., 2017). The dispensing of antibiotics without a prescription may be attributed to the weak enforcement of the medicine’s regulations and the unethical financial motivation of business owners (Parulekar et al., 2016). To ensure the safety of pregnant women, pharmacy professionals should be sensitised to the potential dangers of these medicines, and national medicine regulators should restrict the dispensing of antibiotics without a prescription.

The study's findings showed that one-fourth of women who self-medicated with CMs were exposed to potentially risky medicines, such as ibuprofen which can cause miscarriage, foetal renal impairment, delayed onset of labour, and prolongation of bleeding time in mothers (Antonucci et al., 2012). Although artemether-lumefantrine is categorised as potentially risky, it is the first-line treatment for uncomplicated malaria in pregnancy in Sierra Leone and many other countries, based on the WHO recommendations (WHO, 2022). Pregnant women should consult their doctors or pharmacists before taking any medication to minimise the potential risks of self-medication. Pregnant women self-medicated with herbs such as *Luffa acutangula* and *Citrus aurantifolia* for common ailments such as oedema and vomiting. Luffa acutangula is commonly used in Sierra Leone because of its folkloric therapeutic benefits in infertility, oedema, and urinary tract infections (James et al., 2018a; James et al., 2018b). However, animal studies have shown that they have abortifacient effects, harmful effects on female reproductive organs, and foetal development (Solomon et al., 2021; Yeung et al., 1991). Therefore, pregnant women should be educated and sensitised about the merits and demerits of herbal medicine.

In our study, women with higher education and income were more likely to engage in self-medication, a finding that aligns with previous research (Alonso-Castro et al., 2018; Shokrzadeh et al., 2020). However, this result contradicts the notion that low income and education are typical predictors of self-medication (Lutz et al., 2020; Marwa et al., 2018). The findings from this study could be because women with higher education and income could read and obtain information about medicines from diverse sources such as the internet, thinking that they can manage mild illness without consulting a healthcare professional. Although this practice might seem suitable, it could have severe public health implications such as adverse pregnancy complications. It is always advisable for pregnant women to consult healthcare professionals, such as physicians or pharmacists, for appropriate guidance on the use of medicines and to ensure the well-being of both the mother and the baby. Pregnant women without perceived maternal illnesses were less likely to resort to self-medication compared to those with health issues, suggesting that women with health issues may self-medicate due to past experiences or beliefs about the effectiveness of the products (Zewdie et al., 2018).

### Strengths and limitations of the study

Our study has several advantages. Firstly, it is the first to focus on self-medication among pregnant women in Sierra Leone, a country with one of the highest maternal mortality ratios globally, which is unique and provides valuable insights to target interventions. Secondly, an appropriate sample size was calculated, ensuring precise estimates of self-medication prevalence. Lastly, we followed strengthening reporting of observational studies (STROBE) guidelines, resulting in a robust study report.

However, our research also has limitations. As a cross-sectional study, we cannot draw causal conclusions about self-medication and associated factors. Additionally, our study sites were limited to a few hospitals, restricting generalizability. A non-probability sampling method was employed which has the proclivity to skew the findings. The interviewer-administered response mode could have introduced response bias, and recall bias may have affected the pregnant women's recollection of information. Lastly, we did not assess the safety profile of HMs.

## Conclusion

Self-medication with CMs and/or HMs is common among pregnant women, and most of the CMs and HMs were considered probably safe and contraindicated respectively. The main reason for self-medication was previous disease experience and the predictors were high monthly income, higher education level, and perceived illness. Addressing this issue requires increased education efforts and stricter policies on the sale and distribution of medication without prescriptions. Future research should assess the safety and standardisation of HMs used in pregnancy.

## Data Availability

Data used in this investigation is available at https://doi.org/10.5281/zenodo.10938645.
